# Supplementary material for: Discordance Between Textual Reasoning and Visual Interpretation in Large Language Models for Low Back Pain: Cross-Sectional Quantitative Evaluation and Exploratory Multimodal Stress Test
Source: JMIR Med Inform. 2026 Aug 20;14:e93522. doi: 10.2196/93522 (PMC13494679; doi:10.2196/93522)
Supplement: Supplementary file 1 [file medinform-v14-e93522-s001.docx]

**Appendix 1**

This appendix provides the complete details of the exploratory multimodal stress test (N=5) discussed in the manuscript. For each case, we present the de-identified clinical images, the exact structured prompts used to query the Large Language Models (LLMs), and the gold standard reference answers established by our multidisciplinary expert panel for scoring purposes. Additionally, it should be noted that since some of the original axial images lacked specific annotations for the corresponding intervertebral disc segments, we added the relevant segment labels to these affected images（e.g.L3-4).

Q26

Prompts：Act as an experienced radiologist. I am providing you with several key imaging slices from a patient with low back pain; please carefully identify and analyze these uploaded images. Strictly following the format of a standard radiological report, please provide a complete description of the imaging findings (such as the observed anatomical structures and any abnormal signals or signs), and state your final radiological diagnosis.

De-identified copies:

Gold Standard Reference:

Imaging Description:

Unenhanced sagittal and coronal lumbar scans show: Normal lumbar lordosis is maintained. Marginal hyperostosis and osteophyte formation are observed in all vertebral bodies. A localized depression is noted at the inferior endplate of the L4 vertebral body. No obvious intervertebral space narrowing is observed. Facet joints show hypertrophic sclerosis. Axial scans show: Vacuum phenomenon in the L4/5 and L5/S1 intervertebral discs. L3/4 disc bulging accompanied by left posterior herniation, compressing the thecal sac and left nerve root, with bilateral lateral recess stenosis and no obvious thickening of the bilateral ligamentum flavum. L4/5 and L5/S1 disc bulging circumferentially, compressing the thecal sac; the effective volume of the spinal canal at the L4/5 level is slightly narrowed, with bilateral nerve roots compression, bilateral lateral recess stenosis, and slight thickening of the bilateral ligamentum flavum.

Final Diagnosis:

1. Degeneration of the L4/5 and L5/S1 intervertebral discs.
2. L3/4 disc bulging with herniation (foraminal type); L4/5 and L5/S1 disc bulging; slight spinal canal stenosis at the L4/5 level.
3. Lumbar spine degeneration

Q27

Prompts：Act as an experienced radiologist. I am providing you with several key imaging slices from a patient with low back pain; please carefully identify and analyze these uploaded images. Strictly following the format of a standard radiological report, please provide a complete description of the imaging findings (such as the observed anatomical structures and any abnormal signals or signs), and state your final radiological diagnosis.

De-identified copies:

Gold Standard Reference:

Imaging Description: Lumbar scoliosis with straightened lordosis is observed. The osseous structures of all vertebral bodies remain intact. Marginal osteophyte formation is noted across the vertebral bodies. The L5/S1 intervertebral space is narrowed, accompanied by roughness at the adjacent vertebral endplates. No obvious narrowing is seen in the remaining intervertebral spaces, and the surrounding soft tissues show no obvious abnormalities. Axial scans reveal circumferential bulging of the L3/4 and L4/5 intervertebral discs, which compress the thecal sac and abut the bilateral nerve roots; no obvious thickening of the bilateral ligamentum flavum is observed. The L5/S1 intervertebral disc herniates posteriorly, abutting the thecal sac without obvious compression of the bilateral nerve roots; no obvious thickening of the bilateral ligamentum flavum is noted. Incidental finding: Localized increased bone density at the articular surfaces of the bilateral sacroiliac joints.

Final Diagnosis:

1. L3/4 and L4/5 intervertebral disc bulging; L5/S1 intervertebral disc herniation (central type).
2. Lumbar spine degeneration with slight scoliosis.
3. Endplate inflammation (Modic changes) at the adjacent vertebral margins of L5/S1.
4. Incidental finding: Osteitis condensans ilii of the bilateral sacroiliac joints.

Q28

Prompts：Act as an experienced radiologist. I am providing you with several key imaging slices from a patient with low back pain; please carefully identify and analyze these uploaded images. Strictly following the format of a standard radiological report, please provide a complete description of the imaging findings (such as the observed anatomical structures and any abnormal signals or signs), and state your final radiological diagnosis.

De-identified copies:

Gold Standard Reference:

Imaging Description: Normal lumbar lordosis is maintained. The osseous structures of all vertebral bodies remain intact. Anterior translation of the L5 vertebral body is observed, exceeding 1/4 of the width of the underlying vertebral body. There are bilateral pars interarticularis defects at L5, with non-union of the L5 vertebral arch and irregular osseous margins. No obvious narrowing is observed in the intervertebral spaces, and the surrounding soft tissues show no obvious abnormalities. Non-union of the sacral spinous processes is noted. Axial scans reveal posterior bulging of the L4/5 and L5/S1 intervertebral discs, compressing the thecal sac and local nerve roots, resulting in spinal canal stenosis at the corresponding levels. No obvious bulging or herniation is seen in the remaining visualized intervertebral discs.

Final Diagnosis:

1. L4/5 and L5/S1 intervertebral disc bulging accompanied by spinal canal stenosis at the corresponding levels.
2. Grade II anterior spondylolisthesis of L5; bilateral spondylolysis (pars interarticularis defects) at L5.
3. Non-union of the L5 vertebral arch with irregular osseous margins; further evaluation with MRI is recommended.

Q29

Prompts: Act as an experienced medical specialist. The patient is a 35-year-old female complaining of low back pain due to improper posture, with occasional radiating pain and numbness in the left lower extremity. The pain is primarily localized to the left L3 transverse process and is aggravated by exertion, with no bowel or bladder incontinence. Physical examination reveals: lumbar lordosis is not significantly straightened; lumbar muscle tension is present, with palpable cord-like nodules in the left lumbar musculature; slightly restricted lumbar rotational mobility. L2-L5 paravertebral tenderness (-), left percussion tenderness (-), left lower extremity radiating pain (±), left gluteus medius tenderness (±). Bilateral straight leg raise (SLR) test (-), Bragard's reinforcement test (-), neck flexion test (-), abdominal compression test (-), bilateral FABER (Patrick's) test (-), bilateral piriformis tension test (-), bilateral Ely's test (±). Muscle strength and tone are normal in all four extremities, and bilateral Babinski sign is (-). I have uploaded the key images for this patient. Please integrate the clinical text above with the uploaded imaging evidence to provide a comprehensive list of the most likely clinical diagnoses (explicitly differentiating between the primary and secondary diagnosis), and recommend the next-step treatment plan based on international clinical guidelines.

De-identified copies:

Gold Standard Reference:

Primary Diagnosis: L3 transverse process syndrome

Secondary Diagnoses: Lumbar muscle strain / Thoracolumbar myofasciitis; Lumbar spine degeneration (with mild L5/S1 disc protrusion); Left gluteus medius myofasciitis.

Q30

Prompts: Act as an experienced medical specialist. The patient is a 37-year-old female presenting with back pain and morning stiffness for 3 years, exacerbated with restricted mobility for 6 months. Three years ago, she developed lumbosacral pain without obvious cause, presenting as continuous aching that was most pronounced at night and in the morning, partially relieved by activity. Initially assuming it was “lumbar muscle strain”, she self-medicated with Ibuprofen with temporary relief. Two years ago, the pain spread, accompanied by alternating bilateral buttock pain, occasionally radiating to the posterior left thigh, aggravated by bending or turning, with no significant relief after rest. Six months ago, symptoms worsened with restricted spinal mobility. There is no fever, rash, or oral ulcers. She has lost approximately 3kg since onset, with normal bowel and bladder function. There is no history of trauma or tuberculosis. Family history notes her father has a history of “back pain” (specifics unknown). Physical examination reveals: restricted lumbar flexion, reduced extension and lateral bending; Schober's test (+), Occiput-to-wall test (-). Sacroiliac joint tenderness (+), percussion pain (±), left FABER (Patrick's) test (+), bilateral SLR test (-), Bragard's reinforcement test (±), right FABER test (-), pelvic compression test (+). Chest expansion is normal. Auxiliary examinations show: Sacroiliac DR indicates “mild proliferation and sclerosis of bilateral SI joint surfaces, narrowed left ischiofemoral space”; routine blood test shows elevated WBC and neutrophils; HLA-B27 positive; ESR 45 mm/h, CRP 10.38 mg/L. I have uploaded the key images for this patient. Please carefully synthesize the history, laboratory results, and the uploaded visual features to provide a comprehensive list of the most likely clinical diagnoses (explicitly differentiating between the primary and secondary diagnosis), and recommend the next-step treatment plan based on international clinical guidelines.

De-identified copies:

Gold Standard Reference:

Primary Diagnosis: Ankylosing spondylitis

Secondary Diagnoses: Lumbar disc herniation; Bilateral renal calculi; Bilateral sacroiliitis.
